# Chronic postoperative endophthalmitis after cataract surgery secondary to vancomycin-resistant *Ochrobactrum anthropi*: case report and literature review

**DOI:** 10.1186/s12348-016-0094-z

**Published:** 2016-07-15

**Authors:** Raageen Kanjee, Anjum F. Koreishi, Angelo P. Tanna, Debra A. Goldstein

**Affiliations:** Department of Ophthalmology, Max Rady College of Medicine, University of Manitoba, M264-99 Cornish Ave., Winnipeg, MB R3C 1A2 Canada; Department of Ophthalmology, Northwestern University Feinberg School of Medicine, 645 North Michigan Avenue, Suite 440, Chicago, IL 60611 USA

**Keywords:** Endophthalmitis, *Ochrobactrum anthropi*, Cataract extraction, Drug therapy, Gram-negative bacterial infection, Drug resistance, Microbial

## Abstract

**Background:**

The aim of this study was to report an unusual case of chronic postoperative endophthalmitis following cataract surgery, secondary to *Ochrobactrum anthropi* that was found to be resistant to vancomycin.

**Findings:**

Anterior chamber paracentesis cultures grew gram negative bacilli *Ochrobactrum anthropi*. The patient was treated with a series of intracameral injections of moxifloxacin, with adjuvant oral moxifloxacin. Posterior sub-Tenon and oral corticosteroids were used to treat cystoid macular edema. Explantation of the intraocular lens (IOL)-capsular bag complex was avoided.

**Conclusions:**

Chronic postoperative endophthalmitis is a rare entity, often due to indolent pathogens that sequester in the capsular bag. Aggressive surgical intervention may be avoided with the use of adequate intraocular antibiotic, provided that the offending organism demonstrates appropriate antibiotic susceptibilities.

## Case

A 60-year-old female underwent uncomplicated bilateral cataract surgery in Colombia, 1 month apart, in 2013. Past ocular history was significant for laser in situ keratomileusis in 2006 and past medical history was unremarkable. One month after cataract surgery in the left eye, vision was reduced to 20/80 with an inflammatory response. After an initial non-contributory anterior chamber paracentesis, she underwent a vitreous tap and intravitreal vancomycin injection without improvement. Two pars plana vitrectomies with intravitreal vancomycin failed to improve vision or inflammation, and all cultures were negative. In January 2014, she returned to the USA and was treated by a retinal specialist, who performed intravitreal and sub-Tenon triamcinolone acetonide injections without improvement and with marked elevation of intraocular pressure (IOP).

## Findings

**Fig. 1:**
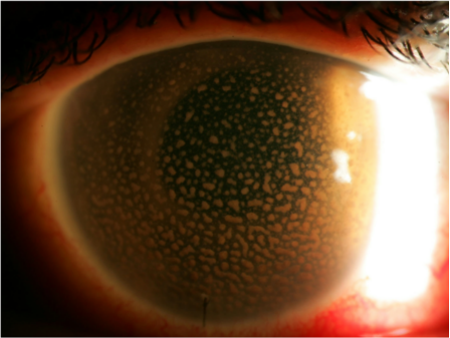
Appearance at presentation: multiple large granulomatous KP

 At referral to a tertiary care uveitis clinic in December 2014, best corrected visual acuity (BCVA) was counting fingers in the left eye and 20/15 in the unaffected right eye. IOP in the left eye, despite maximally tolerated topical and systemic therapy was 42 mmHg. Slit lamp examination demonstrated hundreds of large granulomatous KP and 1+ anterior chamber cell (Fig. 1). Gonioscopy revealed an open angle with no peripheral anterior synechiae, but the view was limited. Fundus examination was limited. Ultrasound biomicroscopy and B-scan of the left eye demonstrated an intraocular lens (IOL) centered in the capsular bag with no retained lens fragments, no significant vitritis, and no retinal detachment.

Intraocular pressure was urgently treated with implantation of an Ahmed FP-7 glaucoma valve (AGV), resulting in postoperative IOP in the mid-teens and improved BCVA of 20/125. An intraoperative sample of aqueous humor was sent for culture and PCR for 16-s eubacterial and 18-s fungal primers, but was not processed due to laboratory error. An initial work-up for sarcoidosis (lysozyme, angiotensin-converting enzyme, chest X-ray), syphilis (*Treponema pallidum* Ab, RPR), and tuberculosis (QuantiFERON®-TB Gold) was negative. Oral prednisone was started 1 day postoperatively.

**Fig. 2:**
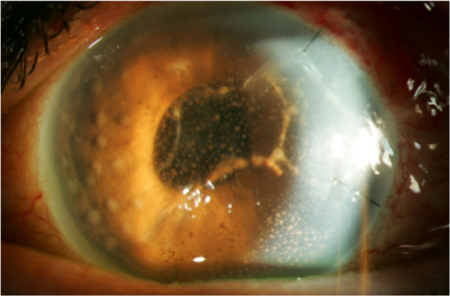
Presentation after initiation of oral steroids post-Ahmed glaucoma valve implantation (superotemporal): new granulomatous iris nodules and anterior chamber fibrin

At the 1-week postoperative follow-up, intraocular inflammation was noted to have worsened, with 3+ anterior chamber cell, new granulomatous iris nodules, and the appearance of golden-brown “strings of pearls” in the anterior chamber (Fig. 2). A 0.3-cm^3^ sample of aqueous humor was again sent for PCR and culture. Intracameral moxifloxacin (160 μg/0.1 cm^3^) was administered, and oral prednisone was tapered. Gram stain revealed gram negative bacilli, and bacterial cultures were positive for *Ochrobactrum anthropi*, resistant to vancomycin, and sensitive to moxifloxacin.

Over the subsequent 3 months, six intracameral moxifloxacin injections (160 μg/0.1 cm^3^, 0.1–0.2 cm^3^) were administered every 1 to 3 weeks based on clinical findings, in conjunction with oral moxifloxacin (400 mg) until aqueous cultures were negative. Oral moxifloxacin was continued at frequencies ranging from daily to three times per week over the subsequent year. Oral prednisone was tapered from 60 mg every 1 to 2 weeks based on clinical response.

**Fig. 3:**
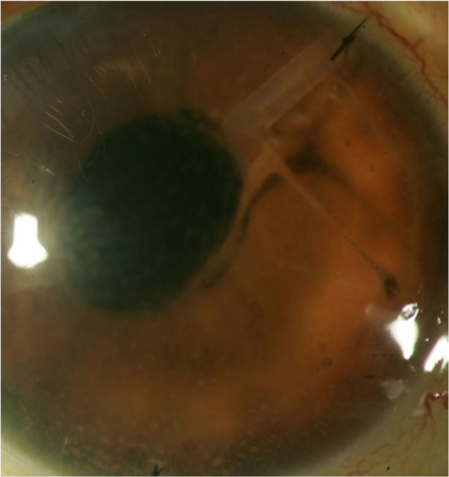
Significant improvement was noted after intracameral moxifloxacin injections. Note the strands were connected to the tube and the suture (*right*)

**Fig. 4:**
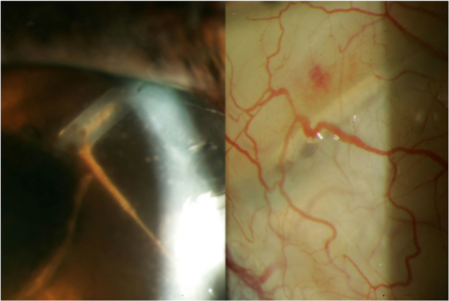
Granulomatous nodules at entrance of Ahmed glaucoma valve tube (*left*), extending toward plate (*right*)

**Fig. 5:**
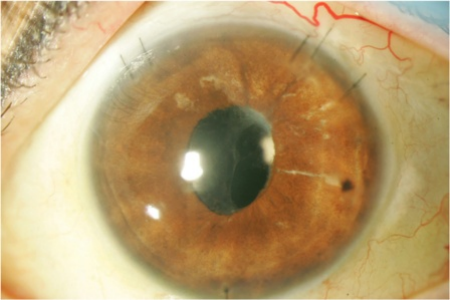
Appearance after removal of Ahmed glaucoma valve

Within 3 months of this treatment regimen, the nodules and fibrin improved (Fig. 3). However, new granulomatous nodules developed along the intracameral portion of the AGV tube and progressed into the subconjunctival portion of the tube, necessitating explantation of the entire device, including the plate (Fig. 4). Cultures from this procedure were negative, and the eye was thoroughly irrigated with moxifloxacin intraoperatively. BCVA improved to 20/50 with a quiet anterior chamber (Fig. 5).

The view of the fundus improved, and the retina appeared normal, with the exception of cystoid macular edema (CME). This was treated with increased oral prednisone and moxifloxacin, and a subsequent series of posterior sub-Tenon triamcinolone injections (0.5–0.6 cc of 40 mg/cc of triamcinolone), after a thorough discussion of the risk of worsening any residual infection. The CME responded well, and oral moxifloxacin and prednisone were tapered. A year after the AGV was removed, IOP was noted to be unacceptably high despite maximally tolerated medical therapy, and a Baerveldt BG 103–250 glaucoma drainage device was implanted, with subsequent improvement in IOP control. As of this writing, BCVA is stable at 20/25 and IOP is 27 on dorzolamide/timolol, atropine, and prednisolone drops. There have been no recurrences 7 months after oral moxifloxacin was discontinued.

## Discussion

*O. anthropi* is an aerobic gram-negative bacillus, first described in 1988 [9]. It is typically found in water sources, both in the community and in hospitals [7]. While not typically pathogenic, *O. anthropi* has been implicated in conditions such as pancreatic abscess and catheter-associated sepsis [2, 7]. It demonstrates a high affinity for implanted medical devices, such as silicone products and other foreign materials [1].

A PubMed literature search (keywords “ochrobactrum anthropi AND (cornea OR cataract OR vitreous OR retina)”) yielded 22 reported cases of ocular infection caused by *O. anthropi*. Another two articles were identified through a similar Web of Science™ search; in total, 23 cases were of chronic postoperative endophthalmitis [3–6, 8, 10, 12, 14, 16] and one case was of microbial keratitis [11]. Of those with endophthalmitis, 19 occurred following cataract surgery [4, 8, 12, 14, 16], one after Boston type 1 keratoprosthesis implantation [5], and three were likely endogenous in origin [3, 6, 10]. For those cases following cataract surgery, the most common treatment involved a combination of vitrectomy with intravitreal antibiotics and capsulectomy with IOL explantation. Antibiotic selection can be challenging, given broad resistance patterns. Table 1 describes reported resistance and sensitivity patterns. In those cases reporting sensitivities, *O. anthropi* has been variably resistant to *β*-lactams, cephalosporins, aminoglycosides, sulfonamides, and trimethoprim. It is usually sensitive to fluoroquinolones and tetracyclines, though sensitivities to other classes have been noted. While visual acuities on presentation have typically been poor (20/100 or worse), with adequate treatment most cases recovered to 20/60 or better (Table 1).

**Table 1:** Antibiotic profiles reported in cases of *O. anthropi* endophthalmitis

Report	Cases	Sensitivities	Resistances	Presenting V_A_	Final V_A_
Braun, [4]	1	Amikacin Ciprofloxacin Imipenem Tetracycline	Cephalosporins Penicillins Sulfamethoxazole Tobramycin Trimethoprim	20/100	20/30
Greven, [8]	1	Ciprofloxacin Imipenem TMP-SMX	Ceftazidime Gentamycin	CF	20/25
Kim, [12]	1	Amikacin Ciprofloxacin Imipenem Meropenem	Aztreonam Ceftazidime Gentamycin Sulperazone Piperacillin	20/100	20/25
Song, [16]	9	Imipenem Quinolones TMP-SMX	Aminoglycosides *β*-lactams	20/200 or worse	20/60 or better
Chiang, [6]	1	Ceftazidime Ciprofloxacin Imipenem Trimethoprim	NR	20/100	20/200
Mattos, [14]	7	NR	NR	NR	NR

*NR* not reported, *TMP-SMX* trimethoprim-sulfamethoxazole, *V*_*A*_ visual acuity

Two epidemics of postoperative endophthalmitis have been reported. Nine cases were attributed to irrigating solution during phacoemulsification in Korea [16], and seven cases were thought to be caused by phacoemulsification machine tubing in Brazil [14]. In two cases, preceding non-ocular surgery with implanted medical devices was noted: one patient with a mitral valvuloplasty [10] and one patient with percutaneous transluminal angioplasty [6].

Chronic postoperative endophthalmitis is a rare entity, significantly less common than acute postoperative endophthalmitis [13]. The most frequently identified bacterial agent is *Propionibacterium acnes*, a gram positive bacillus [15]. Many such indolent pathogens, including *O. anthropi*, may sequester in the capsular bag, necessitating its removal [4, 6, 8, 16].

Prior to aggressive surgical intervention, chronic postoperative endophthalmitis is often treated with intravitreal injections of vancomycin, given its excellent activity against *P. acnes*. It is important to note that in our case, *O. anthropi* was resistant to vancomycin, which contributed to the complexity of management. While initial surgical interventions were unsuccessful, aggressive treatment with intracameral and oral moxifloxacin resulted in a good visual outcome. There was a significant delay in making an accurate diagnosis in this case, despite intraocular infection being investigated at many steps. It is unclear why initial cultures performed in Colombia were negative. Previous reports have suggested that *O. anthropi* should grow on culture media within 6 days of inoculation [16].

As *O. anthropi* has a high affinity for foreign material, there remains concern for its persistent sequestration on implanted medical devices such as IOLs. In the present case, our patient wanted to maintain the IOL if at all possible, and this was achieved with aggressive intraocular and systemic antibiotic therapy. The intraocular inflammation has remained controlled, and CME has not recurred despite discontinuation of oral moxifloxacin. If CME or inflammation recurs, explanation of the IOL and capsular bag will likely be necessary.

While chronic postoperative endophthalmitis continues to be a rare event following cataract surgery, its outcomes may be devastating. *O. anthropi* is a rare causative organism, but should be included in the differential diagnosis, particularly in light of its possible resistance to vancomycin. It may present indolently, similarly to *P. acnes* and atypical mycobacteria. In culture-proven cases with adequate antibiotic sensitivity patterns, a trial of repeated intracameral antibiotic injections and oral antibiotic therapy may control the infection and obviate the need for IOL explantation.

## Abbreviations

AGV, Ahmed glaucoma valve; BCVA, best-corrected visual acuity; CME, cystoid macular edema; IOL, intraocular lens; IOP, intraocular pressure; PCR, polymerase chain reaction; RPR, rapid plasma regain
